# Supplementary material for: Bio-Based and Robust Polydopamine Coated Nanocellulose/Amyloid Composite Aerogel for Fast and Wide-Spectrum Water Purification
Source: Polymers (Basel). 2021 Oct 7;13(19):3442. doi: 10.3390/polym13193442 (PMC8512863; doi:10.3390/polym13193442)
Supplement: Supplementary file 1 [file polymers-13-03442-s001.zip › polymers-1400035-supplementary.pdf]

polymers MDPI

Supplementary Material

Bio-Based and Robust Polydopamine Coated Nanocellulose/ Amyloid Composite Aerogel for Fast and Wide-Spectrum Water Purification

Maxime Sorriaux [1,2], Mathias Sorieul [1] and Yi Chen [1,*]

1 Scion, 49 Sala Street, Private Bag 3020, Rotorua 3046, New Zealand
2 Physico-chimie des Electrolytes et Nanosystèmes InterfaciauX (PHENIX), Sorbonne Université, CNRS, 75005, Paris, France; maxime.sorriaux@sorbonne-universite.fr (M.S.); Mathias.Sorieul@scionresearch.com (M.S.)
* Correspondence: yi.chen@scionresearch.com

Citation: Sorriaux, M.; Sorieul, M.; Chen, Y. Bio-Based and Robust Polydopamine Coated Nanocellulose/ Amyloid Composite Aerogel for Fast and Wide-Spectrum Water Purification. *Polymers* **2021**, *13*, 3442. https://doi.org/10.3390/polym13193442

Academic Editors: Hyun Chan Kim and Lindong Zhai

Received: 14 September 2021
Accepted: 4 October 2021
Published: 7 October 2021

Publisher's Note: MDPI stays neutral with regard to jurisdictional claims in published maps and institutional affiliations.

Copyright: © 2021 by the authors. Licensee MDPI, Basel, Switzerland. This article is an open access article distributed under the terms and conditions of the Creative Commons Attribution (CC BY) license (http://creativecommons.org/licenses/by/4.0/).

Table S1. Source materials used four the CpA aerogel synthesis and the adsorptions experiments.

Chemical	Company
cellulose nanofibrils (3 w%)	American process
amyloid fibrils (6 wt %)	Hi-Aspect Ltd.
dopamine hydrochloride	Sigma–Aldrich
sodium periodate	Sigma–Aldrich
sodium hydroxide	Sigma–Aldrich
nitric acid (60%)	Sigma–Aldrich
Trizma base buffer	Sigma–Aldrich
copper sulphate	Sigma–Aldrich
lead nitrate	Sigma–Aldrich
Rhodamine Blue (RB)	Sigma–Aldrich
Malachite Green (MG)	Sigma–Aldrich
Cristal Violet (CV)	Sigma–Aldrich
Acid Fuchsin (AF)	Sigma–Aldrich
Methyl Orange (MO)	Sigma–Aldrich
Acriflavine (AC)	Sigma–Aldrich
Atrazine	Ici crop care
Bisphenol A	Sigma–Aldrich
Ibuprofen	Sigma–Aldrich

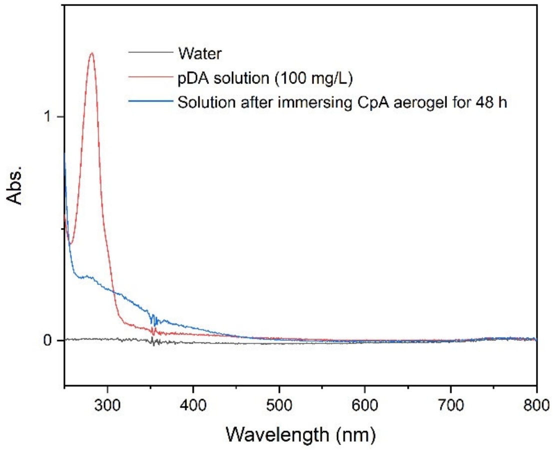

Figure S1. UV-visible spectrum of pure water, pDA solution and the solution after immersing CpA aerogel for 48 h. .

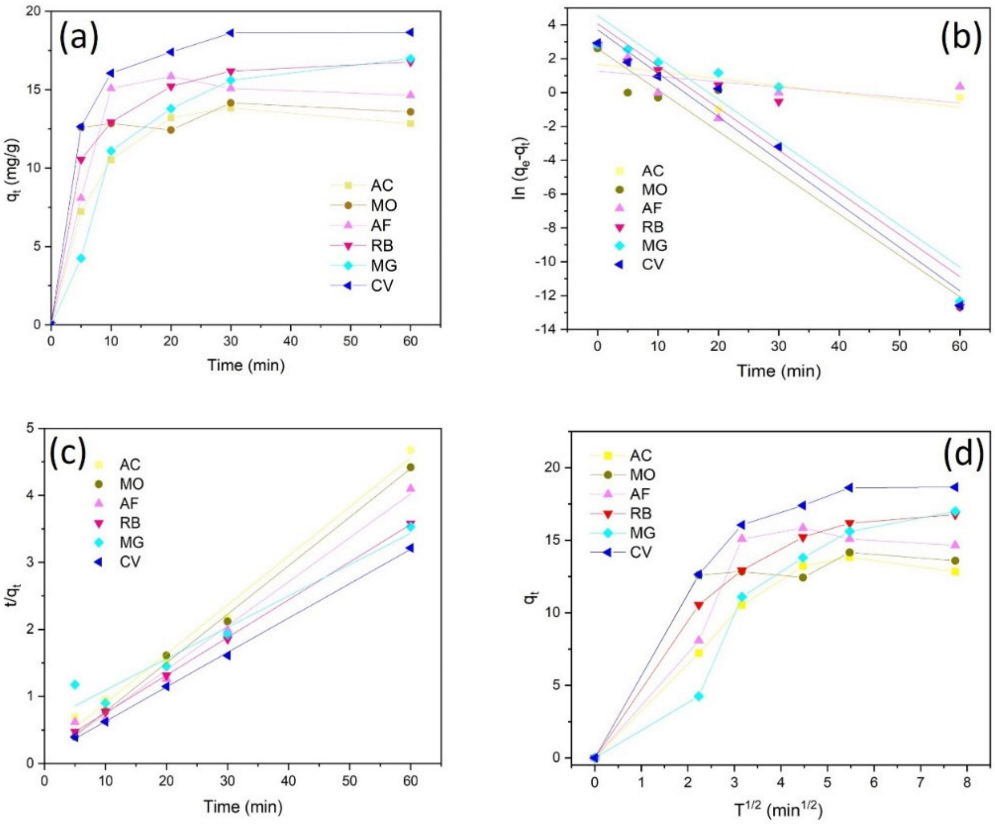

Figure S2. (**a**) Adsorption capability of dyes by the CpA aerogel in function of time. (**b**) The pseudo-first-order kinetic model fitting curve of dyes on the aerogel. (**c**) The pseudo-second-order kinetic model fitting curve of dyes on the aerogel. (**d**) Intra-particle diffusion model for dyes onto the aerogel.

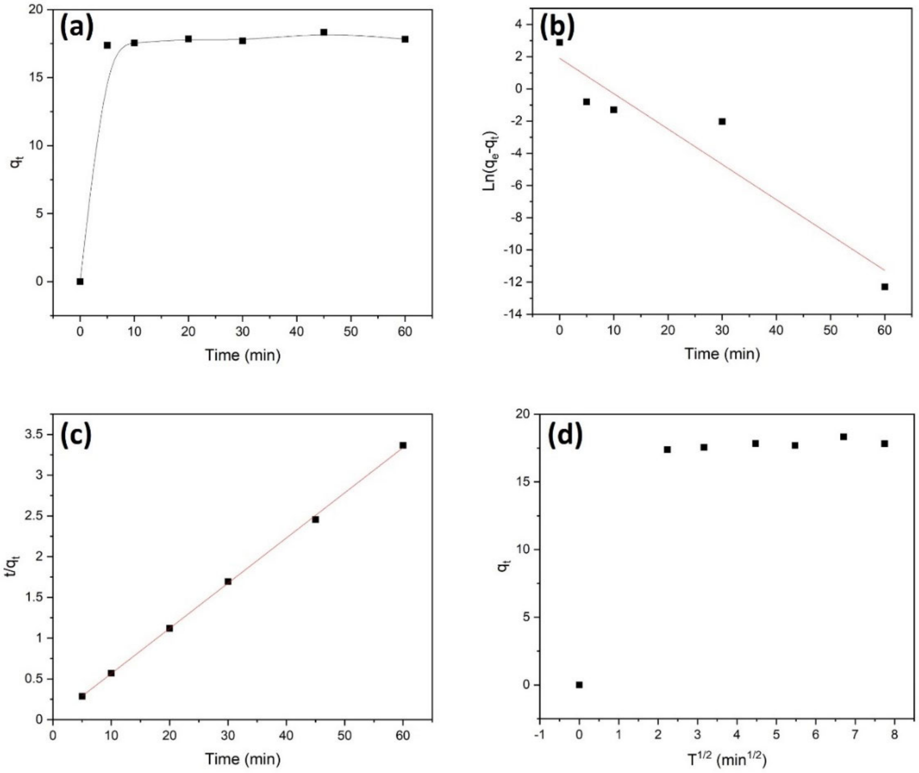

Figure S3. (**a**) Adsorption capability of Pb(II) ions by CpA aerogel in function of time. (**b**) The pseudo-first-order kinetic model fitting curve of Pb(II) ions on the aerogel. (**c**) The pseudo-second-order kinetic model fitting curve of Pb(II) ions on the aerogel. (**d**) Intra-particle diffusion model for Pb(II) ions onto the aerogel.

Table S2. Density and porosity of freeze dried CpA aerogels from freezing at different temperatures.

Frozen temperature (°C)	Density (mg/cm^3)	Porosity (%)
−80	76.024	90.056
−196 (liquid nitrogen)	67.900	91.119

Table S3. Parameters of the pseudo-first-order and pseudo-second-order models for organics adsorption on the aerogel.

	q_e (experimental)		R^2	q_e	k_1
Atrazine	0.0360	y=-0.10899x-5.121	-0.15393		
Bisphenol A	0.0958	y=-0.14667x-3.47869	0.39084		
Ibuprofen	0.0823	y=-0.1541x-3.88557	0.27801		
	**q_e (experimental)**		**R^2**	**q_e**	**k_2**
Atrazine	0.0360	y=27.61217x+13.47533	0.99924	0.0362	763.1024
Bisphenol A	0.0958	y=10.35315x+5.36855	0.99990	0.0965	107.3854
Ibuprofen	0.0823	y=12.09586x+4.00989	0.99994	0.0827	146.214

Table S4. Parameters of the pseudo-first-order model for dyes adsorption on the aerogel.

	q_e (experimental)		R^2	q_e	k_1
AC	13.5826	y=-0.04242x+1.66581	0.29719	5.2900	0.04242
MO	13.5803	y=-0.2443x+2.59156	0.92344	13.3506	0.2443
AF	16.0656	y=-0.03126x+1.25976	-0.00834	3.52458	0.03126
RB	16.7677	Y=-0.24896x+4.06284	0.88097	58.1391	0.24896
MG	16.9858	y=-0.24773x+5.54571	0.84890	256.1364	0.24773
CV	18.6585	y=-0.25706x+3.70838	0.96057	40.7877	0.25706

Table S5. Parameters of the pseudo-second-order model for dyes adsorption on the aerogel.

	q_e (experimental)		R^2	q_e	k_2
AC	13.5826	y=0.07306x+0.17344	0.98796	13.6874	0.0308
MO	13.5803	y=0.0724x+0.05448	0.99684	13.8122	0.0962
AF	16.0656	y=0.06564x+0.08484	0.9877	15.2346	0.0508
RB	16.7677	y=0.05624x+0.19296	0.99975	17.7809	0.0134
MG	16.9858	y=0.04681x+0.62618	0.94479	21.3629	0.0035
CV	18.6585	y=0.05137X+0.11468	0.99921	19.4666	0.0230

Table S6. Parameters of the pseudo-first-order and pseudo-second-order models for Pb(II) ions adsorption on the aerogel.

	q_e (experimental)		R^2	q_e	k_1
pseudo-first-order	17.8254	y=-0.94984x+1.90089	0.86961	6.6918	0.94984
	q_e (experimental)		R^2	q_e	k_2
pseudo-second-order	17.8254	y=0.05545x+0.01146	0.9993	18.0342	0.0031
